# Identification and Evaluation of Reference Genes for Quantitative Analysis of Brazilian Pine (*Araucaria angustifolia* Bertol. Kuntze) Gene Expression

**DOI:** 10.1371/journal.pone.0136714

**Published:** 2015-08-27

**Authors:** Paula Elbl, Bruno V. Navarro, Leandro F. de Oliveira, Juliana Almeida, Amanda C. Mosini, André L. W. dos Santos, Magdalena Rossi, Eny I. S. Floh

**Affiliations:** Departamento de Botânica, Instituto de Biociências, Universidade de São Paulo, São Paulo, Brasil; CSIR-National Botanical Research Institute, INDIA

## Abstract

Quantitative analysis of gene expression is a fundamental experimental approach in many fields of plant biology, but it requires the use of internal controls representing constitutively expressed genes for reliable transcript quantification. In this study, we identified fifteen putative reference genes from an *A*. *angustifolia* transcriptome database. Variation in transcript levels was first evaluated *in silico* by comparing read counts and then by quantitative real-time PCR (qRT-PCR), resulting in the identification of six candidate genes. The consistency of transcript abundance was also calculated applying geNorm and NormFinder software packages followed by a validation approach using four target genes. The results presented here indicate that a diverse set of samples should ideally be used in order to identify constitutively expressed genes, and that the use of any two reference genes in combination, of the six tested genes, is sufficient for effective expression normalization. Finally, in agreement with the *in silico* prediction, a comprehensive analysis of the qRT-PCR data combined with validation analysis revealed that *AaEIF4B-L* and *AaPP2A* are the most suitable reference genes for comparative studies of *A*. *angustifolia* gene expression.

## Introduction

Quantitative analysis of gene expression is important for many fields of biological research and in this regard, quantitative real-time PCR (qRT-PCR) is a popular method for mRNA detection and quantification due to its high sensitivity, reproducibility and high throughput capability [1–3], as well as its wide dynamic range [4–9]. For an accurate and reliable analysis, internal controls must be used to eliminate the experimental noise generated by variations in mRNA amount, reverse transcription efficiency and the co-purification of inhibitory compounds that affect the amplification efficiency [10–13].

Genes involved in basic cellular processes, such as cell structure maintenance or primary metabolism, are often used as internal controls to normalize gene expression between samples [14]. Commonly used examples include 18S ribosomal RNA (*18S*), actin (*ACT*), tubulin (*TUB*), glyceraldehyde-3-phosphate dehydrogenase (*GAPDH*), polyubiquitin (*UBQ*) and elongation factor 1-α (*EF 1-α*) [8,15–17]. However, several reports have described the characterization of other reference genes for use in particular species [8,14,16,18–23]. A reliable constitutively expressed control for qRT-PCR analysis should, by definition, exhibit constant levels of transcript abundance between the cells of different tissues and under different experimental conditions [21]; however, the identification of such control genes can be laborious, especially with species for whom a comprehensive genome sequence is not yet available, such as the Brazilian pine (*Araucaria angustifolia*) [24–25]. This native conifer is currently classified as a critically endangered species [26] and the seeds are recalcitrant to storage, since they maintain high levels of water and active metabolic rates at the mature stage, resulting in a rapid loss of viability, so conservation strategies are restricted [27]. The establishment of a successful somatic embryogenesis system as an alternative propagation approach for conservation requires extensive knowledge of the expression profiles of genes related to morphogenesis in the zygotic counterpart. Recently, a comparative transcriptome analysis of Brazilian pine early somatic embryo formation and seed development was reported, providing a foundation for further gene expression studies [25].

This current study describes a survey of the *A*. *angustifolia* transcriptome data set [25], the identification of fifteen potential qRT-PCR constitutively expressed reference genes, and an assessment of their suitability as controls for transcript profiling of a large set of biological samples representing different seed developmental stages, vegetative tissues and embryogenic cell lines.

## Materials and Methods

### Plant material

**Fig 1:**
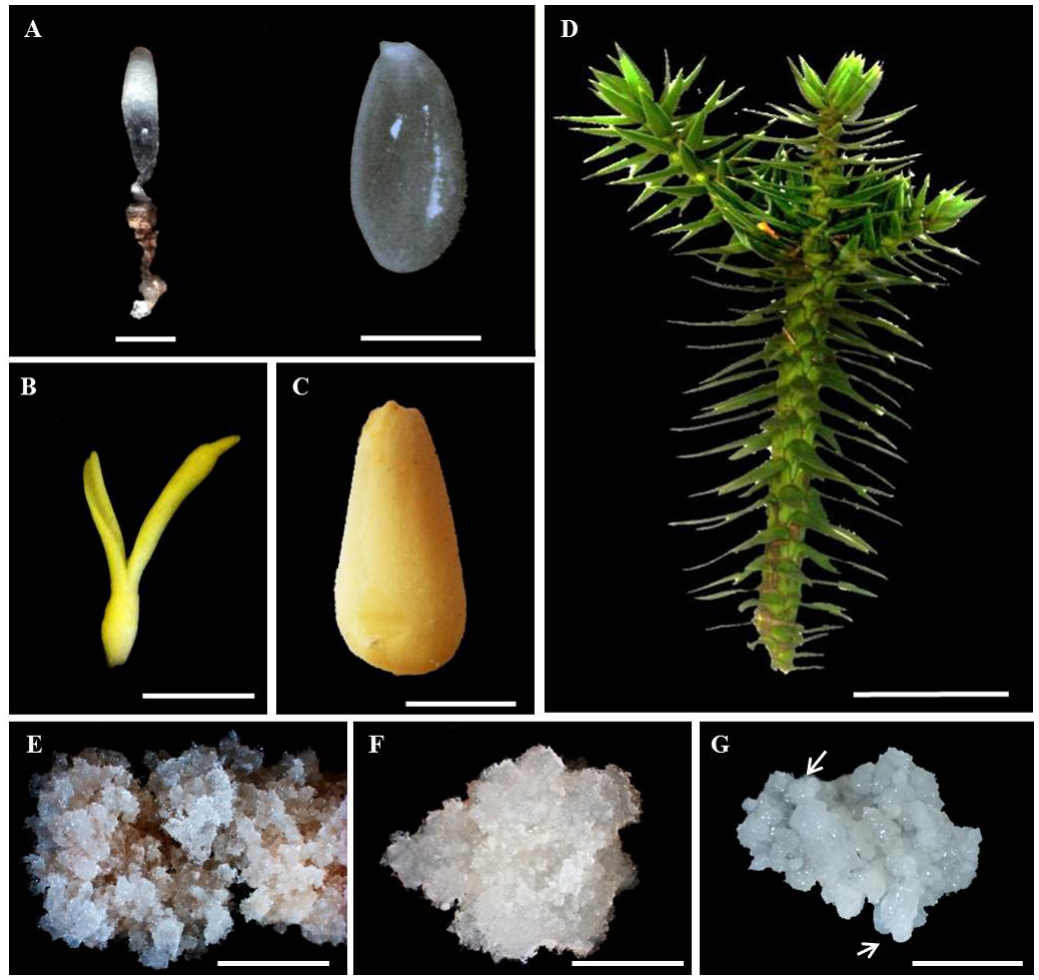
*Araucaria angustifolia* tissues/organs used in this study. Globular zygotic embryos (left, scale bar = 1 mm, and the megagametophyte (right, scale bar = 10 mm) (A); late cotyledonal zygotic embryo (B) and the corresponding megagametophyte (C); aciculas (D); abscisic acid (ABA)-responsive (E), ABA non-responsive (F) and mature ABA-responsive (G) embryogenic cell lines. Arrows indicate globular somatic embryos. Scale bars for panels b-g = 10 mm, 10 mm, 100 mm, 10 mm, 10 mm and 10 mm, respectively.

Two developmental stages of the zygotic embryo (Fig 1A–1C), aciculas (Fig 1D) and three embryogenic cultures of *A*. *angustifolia*, a critically endangered species [26], were analyzed (Fig 1E–1G). The globular zygotic embryos with the corresponding megagametophyte (GZE), cotyledonal zygotic embryos (CZE), megagametophytes of the cotyledonal embryos (CZE MG) and aciculas were harvested from five trees located in the Parque Estadual de Campos do Jordão (22° 41.792' south; 045° 29.393' west, 1.529 m) (authorization by Secretaria do Meio Ambiente, Instituto Florestal, in accordance with CARTA COTEC n° 066/2014 D139/2013 AP), Campos do Jordão, São Paulo, Brazil. Two of the embryogenic cell lines, SE1 and SE6, are abscisic acid (ABA)-responsive and non-responsive, respectively, while the third embryogenic culture was derived from the maturation of embryos generated by SE1 (S1M) (Fig 1). For our studies, the SE1 and SE6 cultures were allowed to proliferate for 21 days on basic MS medium [28] supplemented with 1.46 g dm-3 L-glutamine (MSG) [29] before harvesting. The S1M embryogenic culture was developed by growing the SE1 culture for 90 days on maturation medium (MSG medium supplemented with abscisic acid (ABA), maltose and PEG 4000) and sub-culturing every 30 days [25]. For each zygotic embryo developmental stage, we collected two or three seed cones per mother tree, mixed the seeds and divided them in three sub-samples containing 70 seeds. Each sub-sample was considered a biological replicate. For somatic sample, we collected five callus with 500 mg each (collected from three Petri dishes), mixed, divided in three biological replicates and stored at—80°C until further processing.

### RNA extraction, DNAse treatment and cDNA synthesis

Plant material was homogenized in liquid nitrogen with a pestle and mortar and RNA samples were extracted using three different protocols, depending on the tissue: Chang et al. [30] for zygotic samples, Salzmann et al. [31] for aciculas and Trizol reagent (Invitrogen Life Technologies—Burlington, ON, Canada) for the cultured cell lines. The quantity and purity of the RNA samples were assessed using a Nanodrop ND-100 spectrophotometer (Thermo Fisher Scientific) and, samples with 260/280 nm and 260/230 nm ratios between 1.8–2.2 and 1.6–2.2, respectively, were considered to be of sufficient purity. The integrity of the samples was confirmed by electrophoresis on a 1% (w/v) agarose gel and stained with ethidium bromide. Approximately 2 μg of each RNA sample was subjected to DNAse I (Life Technologies, Inc) treatment and reverse transcribed with random primers using SuperScript III Reverse Transcriptase (Life Technologies, Inc). To confirm that the reverse transcription reaction had worked and to confirm the absence of genomic DNA contamination, samples were subjected to PCR analysis with a pair of intron-flanking primers for Ubiquitin (*UBI- accession number* GW924714.1), Forward: 5’-CCTCGTGTCGATTTACGTC-3’, Reverse: 5’-GGGCGGCTTCTGGATTTG-3’). The PCR reactions were conducted in a total volume of 25 μl containing 1 μl cDNA (1:10), 0.2 μl Taq polymerase (Invitrogen, Carlsbad, CA, USA), 0.2 mM each dNTP, 0.2 μM each primer, 5 μl 10x PCR buffer (Invitrogen, Carlsbad, CA, USA) and 1.5 mM MgCl_2_. The amplification conditions were: 94°C for 3 min, 35 cycles of 94°C for 30 s, 60°C for 30 s, and then 72°C for 1 min. cDNA samples were diluted 1:10 to a final concentration of 5 ng reverse-transcribed RNA/μl.

### Candidate gene selection and primer design

A total of fifteen candidate reference genes were selected, based on the reports of Brunner et al. [32], Iskandar et al. [33], Czechowski et al. [16], Expósito-Rodríguez et al. [18], Hu et al. [34], Cruz et al. [20], Artico et al. [21], Narsai et al. [35], Mafra et al. [36]; de Vega-Bartol et al. [22] and Perini et al. [14] (Table 1). The *A*. *angustifolia* transcriptome database [25] was surveyed using the BLAST program [37] and the corresponding *Arabidopsis thaliana* protein sequences as query sequences. The abundance of the candidate gene transcripts was assessed using the RPKM values (number of reads that map per kilobase of exon model per million mapped reads for each gene, for each tissue or sample), and pairwise and global comparisons between samples and/or tissues, except aciculas (sample not included in the Brazilian pine transcriptome analysis), were performed using the nonparametric Kruskal-Wallis test (p < 0.05) (R software, version 2.8.0) [38]. Specific primers for qRT-PCR were designed according to the MIQE guidelines (see S1 Table) [39] using the program Oligo Perfect 3.1 (http://tools.lifetechnologies.com) based on the sequences listed in Table 2. Primers were tested in PCR reactions using a pool of all cDNA samples as described above.

**Table 1:** Candidate reference genes and their annotated functions.

Gene abbreviation	Gene name	*Araucaria angustifolia* unigene ^a^	*Arabidopsis thaliana* locus	Protein function ^b^	References
*60S*	60S ribosomal protein L18A-1	comp44885_c0_seq2	At1g29970	Structural constituent of ribosome.	*Saccharum* spp. (Iskandar et al. 2004).
*ARP7*	Actin-Related Protein 7	comp43799_c0_seq2	At3g60830	Cell division.	*P*. *trichocarpa* (Brunner et al. 2004); *Saccharum* spp. (Iskandar et al. 2004); *G*. *hirsutum* (Artico et al. 2010); *P*. *abies* and *P*. *pinaster* (de Vega-Bartol et al. 2013); *M*. *domestica* (Perini et al. 2014).
*CYP*	Cyclophilin	comp39853_c0_seq1	At2g21130	Peptidyl-prolyl cis-trans isomerase activity, involved in protein folding.	*P*. *trichocarpa* (Brunner et al. 2004); *G*. *max* (Hu et al. 2009); *C*. *sinensis* (Mafra et al. 2012).
*EF-1α*	Elongation Factor 1α	comp52960_c0_seq8	At5g60390	Calmodulin binding protein involved in translational elongation.	*Arabidopsis thaliana* (Czechowski et al. 2005); *Solanum lycopersicum* (Expósito-Rodríguez et al. 2008); *Gossypium hirsutum* (Artico et al. 2010); *Oryza sativa* (Narsai et al. 2010); *Citrus sinensis* (Mafra et al. 2012); *Picea abies* and *Pinus pinaster* (de Vega-Bartol et al. 2013); *M*. *domestica* (Perini et al. 2014).
*EIF4B-L*	Translational initiation factor 4B	comp50365_c0_seq1	At4g38710	Protein transduction initiation.	*P*. *trichocarpa* (Brunner et al. 2004); *O*. *sativa* (Narsai et al. 2010).
*FBOX*	F-BOX family protein	comp48365_c0_seq1	At5g15710	Cell cycle regulation.	*A*. *thaliana* (Czechowski et al. 2005); *G*. *hirsutum* (Artico et al. 2010); *C*. *sinensis* (Mafra et al. 2012).
*PP2A*	Protein phosphatase 2A	comp39762_c0_seq1	At1g59830	Catalytic subunit of protein phosphatase 2A.	*A*. *thaliana* (Czechowski et al. 2005); *C*. *arabica* (Cruz et al. 2009); *G*. *hirsutum* (Artico et al. 2010); *M*. *domestica* (Perini et al. 2014).
*PSAB*	D1 subunit	comp50019_c0_seq1	ATCG00340	Component of the photosystem I and II reaction centers	*Coffea arabica* (Cruz et al. 2009).
*S24*	S24 ribosomal protein S24	comp54446_c0_seq1	At3g04920	Structural constituent of ribosome.	*C*. *arabica* (Cruz et al. 2009).
*SAM*	S-adenosyl-L-methionine-dependent methyltransferase	comp43283_c0_seq3	At2g32170	Methylation and nucleotide biosynthetic process.	*C*. *sinensis* (Mafra et al. 2012).
*TUA5*	Tubulin α3 / α5 chain	comp14778_c0_seq1	At5g19780	Structural constituent of cytoskeleton.	*Populus trichocarpa* (Brunner et al. 2004); *Glycine max* (Hu et al. 2009); *Malus domestica* (Perini et al. 2014).
*UBC21*	Ubiquitin-conjugating enzyme 21	comp42656_c0_seq3	At5g25760	Fatty acid beta-oxidation, peroxisome organization and phosphatidylinositol biosynthetic process.	*C*. *sinensis* (Mafra et al. 2012); *P*. *abies* and *P*. *pinaster* (de Vega-Bartol et al. 2013).
*UBC9*	Ubiquitin conjugating enzyme	comp52968_c0_seq1	At4g27960	Ubiquitin-dependent protein catabolic process.	*Saccharum* spp. (Iskandar et al. 2004); *C*. *sinensis* (Mafra et al. 2012).
*UBQ7*	Ubiquitin 7	comp51531_c0_seq6	At2g35635	Ubiquitin-dependent protein catabolic process.	*P*. *trichocarpa* (Brunner et al. 2004); *C*. *sinensis* (Mafra et al. 2012).
*UBQ10*	Polyubiquitin	comp51531_c0_seq24	At4g05320	Ubiquitin-dependent protein catabolic process.	*P*. *trichocarpa* (Brunner et al. 2004); *Saccharum* spp. (Iskandar et al. 2004); *A*. *thaliana* (Czechowski et al. 2005); *G*. *max* (Hu et al. 2009); *G*. *hirsutum* (Artico et al. 2010); *O*. *sativa* (Narsai et al. 2010); *M*. *domestica* (Perini et al. 2014).

^a^ *Araucaria angustifolia* transcriptome database (Elbl et al. 2015).

^b^ Encoded-protein function according to TAIR database (http://www.arabidopsis.org/).

**Table 2:** Primers used for gene amplification.

Gene	Primer sequences (forward/reverse primer)	Expected amplicon size (bp)
*Aa60S*	5'-CCTATGTGTGCTTAGATGACC-3'	214
	5'-CCTATTGTTTCCTCTCCTCTCC-3'	
*AaADC* ^a^	5’-GGTGGAGGGCTTGGCATC-3’	199
	5’-CGAAAACGAGGAGGGAATGG-3’	
*AaARP7*	5'-CGGTGTTTTCCAGAAGTTGTCGC-3'	220
	5'-CAGATTGCCTATGAAGAGACGC-3'	
*AaCAT* ^a^	5’-GCTTTTGGAGGACTATCACC-3’	192
	5’-GAGAATCGCACAATAACGGG-3’	
*AaCYP*	5'-GAAAGTTGTTGTTGAAGATTGCGGC-3'	153
	5'-CGTAAACCCTCACAGTAGAAAACC-3'	
*AaEF-1α*	5'-GATGACGATGATGAGGTTTTACTG-3'	164
	5'-CGGCATAATGATTCCACAGC-3'	
*AaEIF4B-L*	5'-CAGTCGCCTCCTGTCTTG-3'	233
	5'-CCGTCGTCTGGTGAAAATG-3'	
*AaFBOX*	5'-CGTCCCCAAATCTTCTCTTCC-3'	196
	5'-GCAAAAGCGAGTTGTTATCTGATG-3'	
*AaPP2A*	5'-GATGAAGGTCAATGTAGAGGG-3'	178
	5'-GGTGGGGCTTATTTTGCTTTG-3'	
*AaPSAB*	5'-CCTCCTCATCTCTTTAGTTTTC-3'	228
	5'-CCCTTCCTTGTCCTGAATC-3'	
*AaS24*	5'-CCCCAGACCATATTTGTTTTCGGC-3'	185
	5'-CTGTTCTTCCTTTCCTTCATTTGC-3'	
*AaSAM*	5'-CACCTCAACAAAGTCCCC-3'	172
	5'-GAACCAAACTCAAGCACCC-3'	
*AaTPS3* ^a^	5’-CGATGAATGTAGCCCTCACTATGC-3’	178
	5’-CTCAATCCAAATCCAATACCCCAGC-3’	
*AaTUA5*	5'-CGTGAGGTGATGTTAGAGAGAG-3'	213
	5'-CGAATGAAGAAGGCGTTTGC-3'	
*AaUBC21*	5'-CTCTGGTGATAATCGTGGG-3'	185
	5'-CACTGGCAGCAAATGGTTG-3'	
*AaUBC9*	5'-CTCTTGAACTGTAACCCCATTCG-3'	221
	5'-GAAGCCTGCCACCTATGAGC-3'	
*AaUBQ7*	5'-CCAATCCCGAGCCCTTTCAG-3'	232
	5'-CCAGCGAATATAAGCCTCTGC-3'	
*AaUBQ10*	5'-CCAATCCCGAGCCCTTTCAG-3'	232
	5'-CCAGCGAATATAAGCCTCTGC-3'	
*AaUGP* ^a^	5’-GAAGTTGTGGTTCCCTATC-3’	214
	5’-CTCTGCTATTGTATTTGTCGTTGAG-3’	

^a^ Genes used for reference gene validation.

### qRT-PCR analysis

Transcript abundance was assessed by qRT-PCR analysis using a 7500 Real-Time PCR system (Applied Biosystems by Life Technologies, NY, USA) (S1 Table). The PCR reactions were performed with 4 μl of cDNA (1:10), 10 μl 2X SYBR Green Master Mix (Applied Biosystems) and the following cycling conditions: 95°C for 10 min, 40 cycles of 95°C for 15 s, 60°C for 30 s and 72°C for 30 s. All reactions were performed in duplicate for each of three biological replicates. The amplification of single products was confirmed by melting curve analysis. After testing primer concentrations of 200 nM, 400 nM and 800 nM, we selected 400 nM as the optimal concentration based on the lowest quantification cycle (Cq) values and the absence of primer dimers. The Cq values and the efficiency of the reaction with each primer were determined using LinRegPCR software [40] and only the genes with transcripts yielding Cq values ≤ 35 were included in subsequent analyses (S1 Table).

### Statistical analysis of gene expression consistency

The consistency of expression of the candidate reference genes was evaluated according to Expósito-Rodríguez et al. [18], by applying two different statistical approaches: using geNorm v.3.5 software (http://medgen.ugent.be/~jvdesomp/genorm/) [41] and NormFinder software (http://www.mdl.dk/publicationsnormfinder.htm) [42].

### Validation of the selected reference genes

To confirm our procedure for the selection of control genes, the relative expression level of *AaADC*, *AaCAT*, *AaTPS3* and *AaUGP* were evaluated using five combinations of reference genes and the results were confronted with Araucaria transcriptome dataset [25]. The primers (Table 2) and qRT-PCR reactions were conducted as described in qRT-PCR section. The relative expression was quantified in comparison with the average expression, normalized against the aciculas samples, and the Cq values of target genes were normalized against the geometric average of a combination of reference genes, followed by ANOVA analysis. The co-variation between the qRT-PCR genes and the transcriptome profile were calculated as Euclidean distances by the *omeSOM software (version v2.27.17, available in http://sourcesinc.sourceforge.net/omesom/). For this analysis, data were normalized as previously described [43]. A 3 X 3 map was selected to group co-expresssed genes showing direct expression patterns using group neighbor neurons with a visualization neighborhoods equal 1 (Vn = 1).

## Results

### Identification of putative *Araucaria angustifolia* reference genes

Using the sequences of previously reported *A*. *thaliana* orthologs, fifteen putative reference genes were retrieved from the *A*. *angustifolia* transcriptome database. In order to identify suitable constitutively expressed reference genes for a number of distinct tissues, genes from a range of functional categories, such as ‘cytoskeleton and cell division’, ‘protein/lipid/nucleotide metabolism’, ‘photosynthesis’ and ‘gene expression regulation’ were chosen (Table 1). An *in silico* analysis of the *A*. *angustifolia* transcriptome data set indicated that of the candidate genes, only *AaPSAB*, *AaPP2A* and *AaEIF4B-L* showed consistent transcript levels amongst the samples represented in the transcriptome database (Table 3), suggesting that these represented the most promising candidates to be used as constitutively expressed reference genes.

**Table 3:** *In silico* analysis of RPMK average ± standard deviation values of the candidate reference genes retrieved from the *Araucaria angustifolia* transcriptome database (Elbl et al. 2015).

	Sample ^a^						
Gene	GZE	CZE	CZE MG	S1M	SE1	SE6	Kruskal-Wallis (p < 0.05) ^b^
*AaTUA5*	1,353.0 ± 762.0	956.2 ± 28.3	1,052.9 ± 98.0	149.0 ± 54.7	240.8 ± 126.1	240.2 ± 14.1	**0.01**
*AaPSAB*	8.7 ± 12.8	1.0 ± 0.7	3.9 ± 0.8	1.4 ± 0.8	3.7 ± 5.4	1.6 ± 0.3	0.27
*AaEF-1α*	9.0 ± 1.1	16.2 ± 0.5	9.8 ± 0.1	0.6 ± 0.4	3.9 ± 2.1	2.4 ± 0.6	**0.01**
*AaS24*	25.7 ± 8.1	30.7 ± 0.8	29.8 ± 11.9	9.9 ± 6.9	72.6 ± 34.5	94.1 ± 15.1	**0.02**
*AaPP2A*	27.7 ± 3.9	27.6 ± 1.3	33.4 ± 6.1	6.2 ± 4.3	53.4 ± 27.0	35.5 ± 4.5	0.07
*AaCYP*	135.2 ± 52.9	180.6 ± 11.7	121.4 ± 25.2	42.0 ± 11.4	222.9 ± 104.9	1,008.4 ± 198.7	**0.02**
*AaUBC21*	8.7 ± 2.0	6.8 ± 0.9	10.0 ± 0.4	2.3 ± 2.1	26.7 ± 7.4	17.8 ± 2.6	**0.01**
*AaSAM*	2.5 ± 0.4	4.6 ± 0.6	2.5 ± 0.4	0.8 ± 0.4	8.0 ± 4.5	4.2 ± 1.1	**0.01**
*Aa60S*	179.9 ± 33.6	187.7 ± 15.7	195.4 ± 53.4	13.5 ± 8.2	87.2 ± 37.8	135.2 ± 13.3	**0.02**
*AaUBC9*	241.2 ± 63.1	307.4 ± 2.6	314.2 ± 61.4	796.3 ± 432.1	1,377.9 ± 1,281.5	1,807.3 ± 212.4	**0.01**
*AaUBQ10*	76.6 ± 22.7	155.9 ± 10.0	98.4 ± 26.7	63.0 ± 12.7	171.6 ± 60.4	219.7 ± 29.0	**0.01**
*AaARP7*	3.9 ± 1.9	7.4 ± 1.1	3.6 ± 0.2	0.8 ± 1.0	4.9 ± 2.2	2.6 ± 0.9	**0.04**
*AaFBOX*	1.3 ± 0.2	3.7 ± 0.7	2.0 ± 0.3	0.6 ± 0.4	4.8 ± 2.5	2.9 ± 0.3	**0.02**
*AaEIF4B-L*	51.0 ± 37.3	83.0 ± 4.2	49.1 ± 10.2	28.0 ± 12.4	51.8 ± 26.3	83.3 ± 18.5	0.10
*AaUBQ7*	93.3 ± 21.0	182.0 ± 12.7	103.2 ± 26.2	57.2 ± 11.7	251.5 ± 114.2	390.3 ± 145.3	**0.01**

^a^ Samples according to Fig 1.

^b^ Genes with significant differential expression among samples are highlighted in bold.

### cDNA quality and test of primers

**Fig 2:**
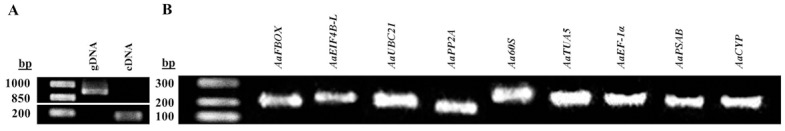
cDNA quality (a) and primer (b) test. Amplification products of PCR analyses using genomic DNA (gDNA) or a pool of all cDNA samples (Fig 1) and *UBI* intron-flanking specific primers (A). Amplicons obtained by PCR using a pool of all cDNA samples and specific primers for the reference genes (B). bp = base pairs.

cDNA samples were derived from RNA extracted from the globular zygotic embryo plus megagametophyte (GZE), the late cotyledonal zygotic embryo (CZE) and the corresponding megagametophyte (CZE MG), the ABA-responsive (SE1) embryogenic cell lines, the ABA-non responsive (SE6) embryogenic cell lines, the maturated SE1 (S1M) embryogenic cell lines and aciculas. All samples were extracted as biological triplicates. The effectiveness of the DNAse treatment was tested by PCR using specific *UBI* intron-flanking primers (Fig 2A).

Out of the fifteen genes initially selected, only nine were successfully amplified from a pool of cDNA (Fig 2B). For *AaS24*, *AaSAM*, *AaUBC9*, *AaARP7* and *AaUBQ7* no amplification products were obtained, while for *AaUBQ10* the amplicon was larger than expected. The might be probably due to errors in transcriptome assembly. The efficiency and specificity of the primer pairs were tested in qRT-PCR. *AaTUA5*, and *Aa60S* showed Cq values ≥ 35 cycles and the reactions did not reach the plateau phase, while the primers for *AaPSAB* resulted in dimer formation. Thus, six genes (*AaFBOX*, *AaEF-1α*, *AaPP2A*, *AaUBC21*, *AaEIF4B-L* and *AaCYP*) were selected for further analyses.

### qRT-PCR amplification

**Fig 3:**
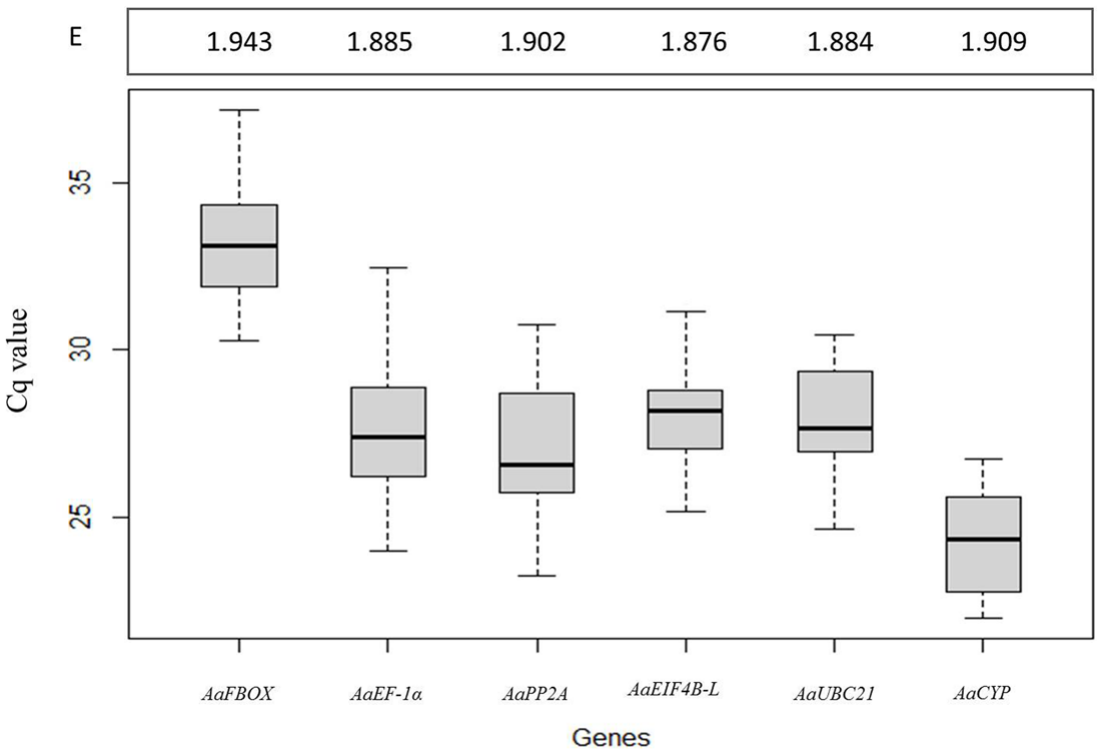
Box plot of the Cq value distribution of candidate reference genes in all *Araucaria angustifolia* samples (Fig 1). The median is indicated by a thick horizontal line. Gray boxes and vertical lines indicate interquartile range and the variance between Cq values for each gene, respectively. E: primer efficiency.

Analysis of the Cq values showed that the selected reference genes had different levels of expression among the analyzed samples, with *AaCYP* (24.30 ± 1.36) and *AaFBOX* (33.27 ± 1.92) being the most highly and least abundantly expressed, respectively. *EF-1α*, *AaPP2A*, *AaEIF4B-L* and *AaUBC21* showed intermediate levels of expression, with average Cq values from 25 to 29 cycles. The intra-assay variation was evaluated by correlation analysis and demonstrated a reasonably good fit (r2 = 0.992, S1 Fig). The efficiency for all six primer pairs was ≥ 1.88 for the entire experimental set of samples (Fig 3).

### Analysis of reference gene expression using geNorm and NormFinder software

The consistency of gene expression was analyzed using geNorm (Table 4) and NormFinder (Table 5) software with data derived from the entire set of samples (ALL), or from four subsets: zygotic (GZE, CZE and CZE MG), cell lines (SE1, SE6 and S1M), zygotic/somatic embryos (GZE and S1M) and aciculas. The geNorm analysis scored all six genes as suitable ‘normalizers’ according to their stability value, although the relative ranking of the genes varied depending on the sample. *AaPP2A*, *AaEIF4B-L* and *AaCYP* exhibited the most consistent expression among the different samples except for the “cell line” subset. The optimal number of reference genes was calculated by the pairwise variation value (*V)* and it was found that for a cut-off threshold of 0.15, two reference genes were sufficient for an accurate normalization in all the experimental data sets evaluated (Table 4). NormFinder software also indicated a distinct ranking of genes but the best pair of normalizers for the five samples were different from those suggested by geNorm. The NormFinder analysis further suggested the best combination of genes using a pairwise comparison approach and for only two subsets of data, zygotic and zygotic/somatic embryos, the selected pair was in agreement with the stability ranking, while for the other three the chosen genes were *AaFBOX* and *AaCYP* (Table 5). The best reference gene, as indicated by both geNorm and NormFinder, was different for each subset of samples; however, when the sample diversity was larger (*i*.*e*. by including all the data), the results indicated that *AaEIF4B-L* represents the best reference gene for *A*. *angustifolia*.

**Table 4:** Ranking of *Araucaria angustifolia* candidate reference genes based on GeNorm analysis.

ALL		ZYGOTIC		CELL LINES		ZYGOTIC/SOMATIC EMBRYOS		*ACICULAS* ^*c*^	
Ranking	*M* ^a^	Ranking	*M* ^a^	Ranking	*M* ^a^	Ranking	*M* ^a^	Ranking	*M* ^a^
*AaEIF4B-L/AaPP2A*	0.284	*AaPP2A/AaCYP*	0.280	*AaFBOX/ AaEF-1α*	0.150	*AaPP2A/AaEIF4B-L*	0.205	*AaUBC21/AaCYP*	0.198
*AaEF-1α*	0.310	*AaEIF4B-L*	0.308	*AaEIF4B-L*	0.225	*AaEF-1α*	0.282	*AaEF-1α*	0.427
*AaUBC21*	0.380	*AaEF-1α*	0.336	*AaPP2A*	0.274	*AaFBOX*	0.306	*AaEIF4B-L*	0.399
*AaCYP*	0.399	*AaFBOX*	0.344	*AaUBC21*	0.393	*AaCYP*	0.311	*AaFBOX*	0.476
*AaFBOX*	0.424	*AaUBC21*	0.360	*AaCYP*	0.450	*AaUBC21*	0.341	*AaPP2A*	0.346
***V*** ^b^		***V*** ^b^		***V*** ^b^		***V*** ^b^		***V*** ^b^	
*V2/3*	0.097	*V2/3*	0.097	*V2/3*	0.085	*V2/3*	0.101	*V2/3*	0.136
*V3/4*	0.104	*V3/4*	0.072	*V3/4*	0.075	*V3/4*	0.064	*V3/4*	0.104
*V4/5*	0.073	*V4/5*	0.061	*V4/5*	0.110	*V4/5*	0.060	*V4/5*	0.083
*V5/6*	0.070	*V5/6*	0.059	*V5/6*	0.086	*V5/6*	0.062	*V5/6*	0.089

^a^ Stability coefficient is the mean of the variation of two internal control genes between an individual and all other tested genes. The most stable gene has the lowest *M* value (cut-off < 1.5).

^b^ Pairwise variation values *Vn/n+1* < 0.15 mean that use of the two most stable genes is sufficient to normalize the expression of a test gene in the corresponding set of samples. *n*: number of genes.

**Table 5:** Ranking of *Araucaria angustifolia* reference genes calculated using NormFinder.

ALL		ZYGOTIC		CELL LINES		ZYGOTIC/SOMATIC EMBRYOS		*ACICULAS*	
Ranking	Stability value ^a^	Ranking	Stability value ^a^	Ranking	Stability value ^a^	Ranking	Stability value ^a^	Ranking	Stability value ^a^
*AaEIF4B-L*	0.730	*AaFBOX*	0.576	*AaPP2A*	0.697	*AaCYP*	0.170	*AaEIF4B-L*	0.639
*AaEF-1α*	0.741	*AaPP2A*	0.624	*AaEF-1α*	0.731	*AaFBOX*	0.260	*AaUBC21*	0.732
*AaPP2A*	0.749	*AaUBC21*	0.663	*AaEIF4B-L*	0.785	*AaEF-1α*	0.371	*AaEF-1α*	0.823
*AaUBC21*	0.807	*AaCYP*	0.671	*AaFBOX*	0.942	*AaPP2A*	0.404	*AaPP2A*	0.846
*AaCYP*	0.919	*AaEF-1α*	0.698	*AaUBC21*	1.016	*AaUBC21*	0.428	*AaCYP*	0.885
*AaFBOX*	1.093	*AaEIF4B-L*	0.727	*AaCYP*	1.164	*AaEIF4B-L*	0.586	*AaFBOX*	1.269
***Best Combination*** ^b^		***Best Combination*** ^b^		***Best Combination*** ^b^		***Best Combination*** ^b^		***Best Combination*** ^b^	
*AaFBOX and AaCYP*	0.390	*AaFBOX and AaPP2A*	0.364	*AaFBOX and AaCYP*	0.253	*AaFBOX and AaCYP*	0.165	*AaFBOX and AaCYP*	0.346

^a^ Genes are ranked according to minimal estimated intra- and intergroup variation. Genes with the lowest stability value have the most stable expression.

^b^ The pair of genes with the highest degree of similarity in their expression profiles.

### Validation of the selected reference genes

**Fig 4:**
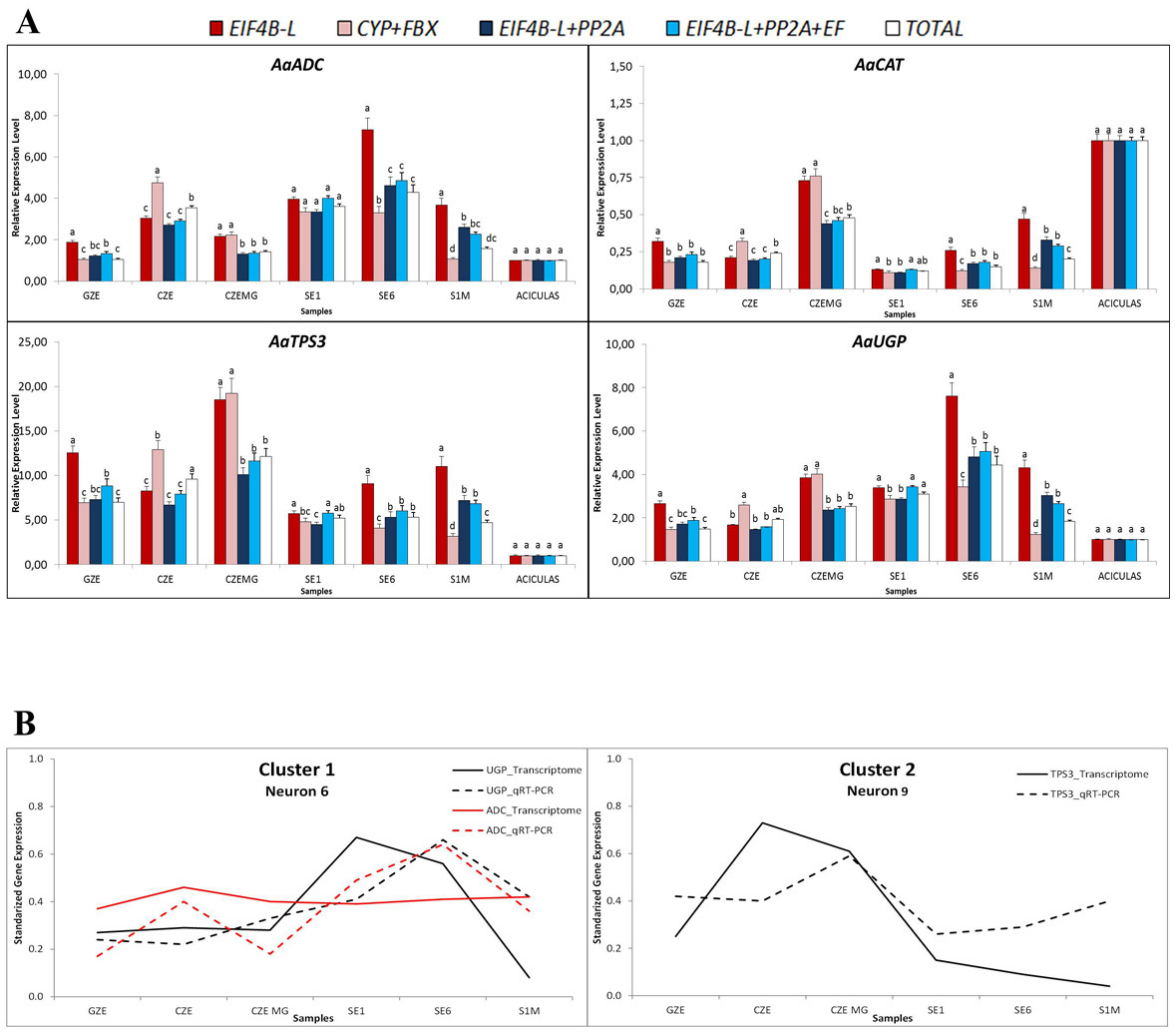
Validation of the most stable reference genes. (A) Relative expression of *AaADC*, *AaCAT*, *AaTPS3* and *AaUGP* in the samples used in this study, normalized with different combinations of reference genes. (B) Co-variation patterns by neural network analysis, performed by applying the *omeSOM software [43].

The relative expression levels of *AaADC*, *AaCAT*, *AaTPS3* and *AaUGP* were used as target genes to demonstrate the usefulness of the candidate reference genes in qRT-PCR. Five combinations of the most stable reference genes established by geNorm and NormFinder were applied for normalization (Fig 4A). In all four situations, when only one normalizer was used (*EIF4B-L*) the relative expression was different than the observed when two or more reference genes were used, except to sample CZE. Likewise, *CYP*+*FBOX* showed discrepant values when compared to *EIF4B-L*+*PP2A* or *EIF4B-L*+*PP2A*+*EF* and similar expression values was observed only for *AaADC* in SE1 and *AaCAT* in GZE. The combinations *EIF4B-L*+*PP2A* and *EIF4B-L*+*PP2A*+*EF* did not exhibited significant difference in expression values, except in SE1 for *AaCAT*, *AaTPS3* and *AaUGP* genes (Fig 4A). The addition of more reference genes did not improve the analysis of target and reference genes.

For comparison between the qRT-PCR and transcriptome profiles we used a neural clustering model through a self-organizing map (*omeSOM) [43], using the qRT-PCR results obtained with the *EIF4B-L*+*PP2A* pair. Among all the genes analyzed *AaADC* and *AaUGP* (Cluster 1) and *AaTPS3* (Cluster 2) showed a correlation between qRT-PCR and transcriptome profile (Fig 4B). The results suggest that normalizers, mainly *EIF4B-L* and *PP2A*, were satisfactory for accurate qRT-PCR assay, since the majority of target genes tested showed relative expression levels correlating with the transcriptome expression profile.

## Discussion

Gene expression analysis is central to developing a mechanistic understanding of physiological and developmental processes and qRT-PCR is currently the most widely used method for quantifying transcript expression [6–7,9,23]. Consequently, the identification of suitable reference genes for use as internal controls to standardize expression levels is an important goal. This is particularly challenging for non-model species that do not have a reference genome sequence [8,14,19–21,23]. The Brazilian pine (*A*. *angustifolia*) is an example of a non-model species for which there is a considerable research invested in studying aspects of growth, development and environmental responses, and thus for which the identification of suitable reference genes would be of considerable value. For example, several studies have investigated the molecular mechanisms that control embryogenesis in *A*. *angustifolia* [44–46] and recently a substantial collection of transcriptome sequences was generated and annotated [25]. Large datasets, such as those associated with microarrays, expressed sequence tags and cDNA libraries, can provide valuable resources to identify genes showing minimal variation in expression [14,24,35]. Accordingly, we used published information derived from several plant species to identify candidate constitutively expressed reference genes in an *in silico* analysis of the *A*. *angustifolia* transcriptome dataset, which contains genes expressed during early somatic embryo and seed development (Table 1). This search suggested fifteen such candidate reference genes and a subsequent non-parametric statistical test highlighted *AaPSAB*, *AaPP2A* and *AaEIF4B-L* as those with invariable expression among the sets of samples that comprise the *A*. *angustifolia* transcriptome dataset (Table 3).

Of the fifteen genes, six were successfully amplified by PCR and showed satisfactory qRT-PCR quality parameters, so their consistency of expression was further evaluated using geNorm and NormFinder software (Tables 4 and 5). According to the former, all the identified genes were deemed sufficiently reliable such that only two should be necessary for an accurate normalization of qRT-PCR data from all the tested subsets of the *A*. *angustifolia* samples.

In accordance with similar studies of gene expression in *Glycine max* [34], *Citrus* spp. [36] and *Pyrus* spp. [47], the best pair of reference genes for *A*. *angustifolia* varied within subsets of samples, suggesting that the genes showing the most constant expression profile varied between tissues and/or samples. It is noteworthy that the two programs suggested different optimal pairs of reference genes for a given subset of samples. However, when all the samples (ALL) were examined together, both algorithms indicated the same three genes as being the most effective for normalizing *A*. *angustifolia* expression data: *AaEIF4B-L*, *AaPP2A* and *AaEF-1α*, which is in agreement with the initial *in silico* analysis. Thus, it appears that the wider and more diverse the sample pool, the more accurate the selection of constitutively expressed genes. We also note that *EF-1α* has been extensively tested as a reference gene and has been ranked as highly effective for use in gene expression studies with *Picea abies* and *Pinus pinaster*, two other gymnosperm species [22]. *EIF4B-L* has not been widely used to normalize gene expression in plants and was reported to be somewhat effective as a reference gene in studies of both vegetative and reproductive organs of *Populus trichocarpa* [32]. Finally, *PP2A* has been shown to have consistent expression levels in *Gossypium hirsutum* and *Malus domestica* leaves, buds, flowers and fruits [14,21]. Thus, for most plant species and samples, there appears to be a suitable group of “universal” candidate reference genes that are constitutively expressed and that encode proteins involved in basic cellular processes (Table 1).

The reference gene approach was validated by quantifying relative expression of *A*. *angustifolia AaADC*, *AaCAT*, *AaTSP3* and *AaUGP* genes. In a previous study, it was observed that transcripts involved in *A*. *angustifolia* embryogenesis related to polyamines, oxidative and carbohydrate metabolism were differentially expressed [25]. *ADC* encodes an enzyme that controls the polyamine flux in seeds of conifers by the putrescine (Put) biosynthesis [48]. Put is one of the main polyamines found in plants and is normally associated to embryogenesis and abiotic stress response [49–50]. Related to stress response, *CAT* plays important roles in plant antioxidative and detoxification processes that are closely related with reactive oxygen species generation [51]. In carbohydrate metabolism, *TPS* and *UGP* have a participation in biosynthesis of plant trehalose (α-D-glucopyranosyl α-D-glucopyranoside) a non-reduced disaccharide which plays important roles in protecting plants from heat, cold, osmotic and dehydration stress [52]. These genes encode enzymes related to sugar sensing, energy metabolism and seem involved in embryogenic potential of *A*. *angustifolia* cell lines (Navarro et al. unpublished data).

For all target genes and samples, similar expression values were observed when normalized with the combinations *EIF4B-L*+*PP2A*, *EIF4B-L*+*PP2A*+*EF* and TOTAL, showing that the use of three or more reference genes is unnecessary (Fig 4A). Interestingly, when we used the *EIF4B-L*+*PP2A* pair in the neural network analysis the result was in accordance with the Araucaria dataset expression profiles (Fig 4B). Therefore, the proposed reference genes are reliable for obtaining accurate expression profiles in different target genes and samples during *A*. *angustifolia* embryogenesis.

In conclusion, this report describes an efficient pipeline for the identification of constitutively expressed reference genes for subsequent gene expression analyses. We also demonstrate that large databases of gene expression can provide valuable resources for identifying genes that show little variation in expression and, in this regard, data derived from a diverse set of samples are recommended for the selection of such reference genes.

## Supporting Information

S1 Fig Intra-assay variation of qRT-PCR. Ct values of the replicates were plotted against each other.
(TIF)

S1 Table MIQE form, qRT-PCR and sample information. (PDF)
