# Supplementary figures and images for: Identification and Evaluation of Reference Genes for Quantitative Analysis of Brazilian Pine (Araucaria angustifolia Bertol. Kuntze) Gene Expression
Source: PLoS One. 2015 Aug 27;10(8):e0136714. doi: 10.1371/journal.pone.0136714 (PMC4552031; doi:10.1371/journal.pone.0136714)

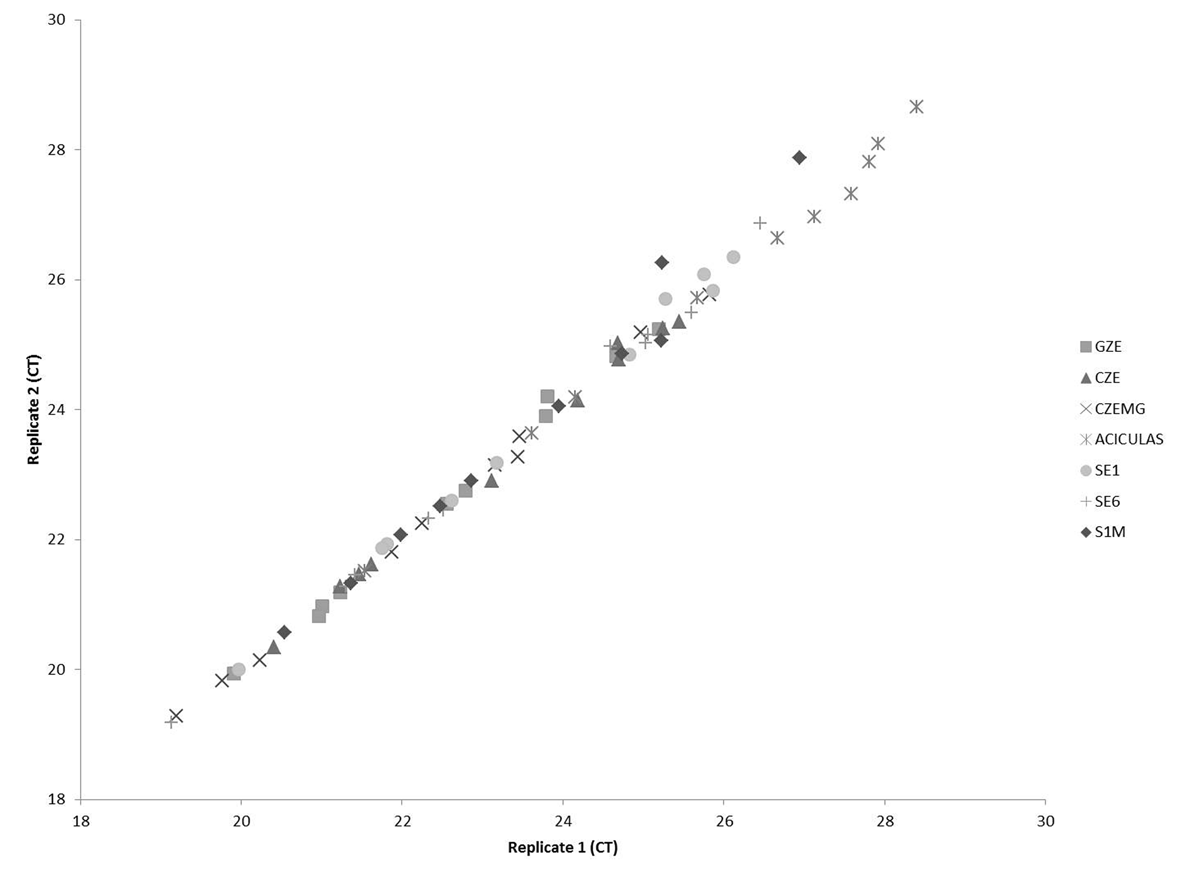

Supplement: S1 Fig — Ct values of the replicates were plotted against each other. (TIF) [file pone.0136714.s001.tif]
