# Supplementary material for: Identification and Evaluation of Reference Genes for Quantitative Analysis of Brazilian Pine (Araucaria angustifolia Bertol. Kuntze) Gene Expression
Source: PLoS One. 2015 Aug 27;10(8):e0136714. doi: 10.1371/journal.pone.0136714 (PMC4552031; doi:10.1371/journal.pone.0136714)
Supplement: S1 Table — (PDF) [file pone.0136714.s002.pdf]

Supplemental Table. MIQE form, qRT-PCR and sample information.

**Experimental design**		**Reference genes array information**
Definition of experimental and control groups	E	*Araucaria angustifolia* seeds in two developmental stages: GZE, CZE and CZE MG. Three embryogenic cultures SE1, SE6, S1M and acicula (leaves).
Number within each group	E	For each zygotic sample, we collected two or three seed cones per mother tree, mixed the seeds and divided them in three sub-samples containing 70 seeds. Each sub-sample was considered a biological replicate. For somatic sample, we collected five callus with 500 mg each (collected from three Petri dishes), mixed, divided in three biological replicates.
Assay carried out by the core or investigator's laboratory?	D	yes
Acknowledgment of authors' contributions	D	-
**Sample**		
Description	E	Globular zygotic embryos with the corresponding megagametophyte (GZE), Cotyledonal zygotic embryos (CZE), megagametophytes of the cotyledonal embryos (CZE MG) and aciculas. Two of the embryogenic cell lines, SE1 and SE6, are abscisic acid (ABA)-responsive and non-responsive, respectively, while the third embryogenic culture was derived from the maturation of embryos generated by SE1 (S1M).
Volume/mass of sample processed	D	see sample information on this file
Microdissection or macrodissection	E	macrodissection
Processing procedure	E	Freeze-dried material tissues were ground to a fine powder in liquid nitrogen
If frozen, how and how quickly?	E	tissue were harvested and immediately frozen into liquid nitrogen
If fixed, with what and how quickly?	E	-
Sample storage conditions and duration (especially for FFPE[2]samples)	E	– 80 ºC until use
**Nucleic acid extraction**		
Procedure and/or instrumentation	E	Total RNA from 1000 mg, 300 mg and 100mg of seeds (GZE, CZE and CZE MG), leaf (aciculas) and embryogenic cell lines (SE1, SE6 and S1M) tissues respectively was isolated using three different protocols, depending on the tissue: Chang et al. (1993) with modifications for seed samples, Salzmann et al. (1999) for aciculas and Trizol® reagent (Invitrogen Life Technologies - Burlington, ON, Canada) for the cultured cell lines.
Name of kit and details of any modifications	E	The extration step of Chang et al. (1993) protocol was replaced by Trizol (Invitrogen) and the purification stage was performed using PureLink RNA Mini Kit (Ambion Life Tecnologies).
Source of additional reagents used	D	-
Details of DNase or RNase treatment	E	DNA traces were removed by treatment with amplification-grade DNAse I (Invitrogen) following the manufacturer instructions
Contamination assessment (DNA or RNA)	E	The suitability of each cDNA for PCR reaction and DNA contamination were checked using an intron-flanking pair of primers of the Ubiquitin *A. angustifolia* gene (UBI- accession number GW924714.1)
Nucleic acid quantification	E	see sample information on this file

Instrument and method	E	NanoDrop spectrophotometer
Purity (A_{260}/A_{280})	D	in general, high-quality samples with OD ratios of at least 1,8 (260/280 nm) and 1,9 (260/230 nm) were used for further analysis; for exceptions see sample information
Yield	D	see sample information sheet on this file
RNA integrity: method/instrument	E	agarose-gel electrophoresis
RIN/RQI or C_q of 3' and 5' transcripts	E	analysis not performed
Electrophoresis traces	D	analysis not performed
Inhibition testing (C_q dilutions, spike, or other)	E	analysis not performed
**Reverse transcription**		
Complete reaction conditions	E	cDNA was synthesised from 1µg of total RNA using Superscript III reverse transcriptase (Invitrogen) according to the manufacturer instructions.
Amount of RNA and reaction volume	E	1 ug in 20 ul
Priming oligonucleotide (if using GSP) and concentration	E	random primers
Reverse transcriptase and concentration	E	Taq DNA polimerase (5U/ul) Invitrogen
Temperature and time	E	according to manufacturer instructions
Manufacturer of reagents and catalogue numbers	D	Invitrogen
C_qs with and without reverse transcription	D[3]	The suitability of each cDNA for PCR reaction and DNA contamination were checked using an intron-flanking pair of primers of the Ubiquitin A. angustifolia gene (UBI- accession number GW924714.1)
Storage conditions of cDNA	D	– 20 ºC
**qPCR target information**		
Gene symbol	E	see primer and amplicon information on this file
Sequence accession number	E	see primer and amplicon information on this file
Location of amplicon	D	data not shown
Amplicon length	E	see primer and amplicon information on this file
In silico specificity screen (BLAST, and so on)	E	blast against NCBI and Araucaria angustifolia transcritome database
Pseudogenes, retropseudogenes, or other homologs?	D	-
Sequence alignment	D	data not shown
Secondary structure analysis of amplicon	D	not done
Location of each primer by exon or intron (if applicable)	E	not done
What splice variants are targeted?	E	analysis not performed
**qPCR oligonucleotides**		**Reference genes array information**
Primer sequences	E	see primer and amplicon information on this file

RTPrimerDB identification number	D	-
Probe sequences	D[4]	none
Location and identity of any modifications	E	none
Manufacturer of oligonucleotides	D	Invitrogen
Purification method	D	none
**qPCR protocol**		
Complete reaction conditions	E	20 µl reaction contained optimized concentration of forward and reverse primer, 10 ul of SYBR mix, cDNA quantity corresponding to 10 or 20 ng of total RNA reverse-transcribed
Reaction volume and amount of cDNA/DNA	E	cDNA samples were 10X diluted to a final concentration of 10 ng reverse-transcribed RNA/ul; 4 ul were used per reaction (see primer and amplicon information)
Primer, (probe), Mg^{2+}, and dNTP concentrations	E	see primer and amplicon information on this file
Polymerase identity and concentration	E	
Buffer/kit identity and manufacturer	E	2X SYBR Green Master Mix (Applied Biosystems)
Exact chemical composition of the buffer	D	-
Additives (SYBR Green I, DMSO, and so forth)	E	-
Manufacturer of plates/tubes and catalog number	D	-
Complete thermocycling parameters	E	10 min at 95ºC, followed by 40 cycles of 15 s at 95ºC, 30 s at 60ºC, and 30 s at 72 ºC. The amplification process was followed by a melting curve analysis
Reaction setup (manual/robotic)	D	manual
Manufacturer of qPCR instrument	E	Applied Biosystems 7500 Real Time PCR System
**qPCR validation**		
Evidence of optimization (from gradients)	D	Optimal concentration of PCR primer pair was selected in a 200–800 nM range based on: lowest Ct values and primer dimer formation
Specificity (gel, sequence, melt, or digest)	E	Specifity was validate by electrophoresis agarose gel and melt curve analysis
For SYBR Green I, C_q of the NTC	E	Ct undected or with more than 5 Cts of diference for the lowest concentration unknown
Calibration curves with slope and *y* intercept	E	-
PCR efficiency calculated from slope	E	The mean PCR efficiency was calculated based on the starting point of the exponential phase of amplification using LinReg PCR program from raw normalized fluorescence as input data (see primer and amplicon information)
CIs for PCR efficiency or SE	D	-
r^2 of calibration curve	E	NA
Linear dynamic range	E	Ct19 to Ct35
C_q variation at LOD	E	NA
CIs throughout range	D	-
Evidence for LOD	E	-
If multiplex, efficiency and LOD of each assay	E	-
**Data analysis**		
qPCR analysis program (source, version)	E	LinRegPCR software (Ruijter et al. 2009)

Method of C_q determination	E	The fluorescence threshold for determining Cq values is automatically set at 1 cycle below the upper limit of the window of linearity
Outlier identification and disposition	E	data not shown
Results for NTCs	E	Ct undected or with more than 5 Cts of diference for the lowest concentration unknown
Justification of number and choice of reference genes	E	Araucaria reference genes described by Exposito-Rodriguez *et al* . (2008) were tested. Analyses performed indicated that the most stable genes were *AaEIF4B-L*, *AaPP2A* as shown in Table 4 and 5
Description of normalization method	E	Normalized gene expression ratios (R) were calculated by the ΔΔCt method with the improvements proposed by Hellemans *et al* . (2007). This algorithm allows using several reference genes at the same time leading to a more accurate relative expression data according to the following equation: $$R = \frac{E_{goi}^{\Delta Ct,goi}}{\sqrt[f]{\prod_{0}^{f} E_{ref_0}^{\Delta Ct,ref_0}}}$$
Number and concordance of biological replicates	D	three
Number and stage (reverse transcription or qPCR) of technical replicates	E	For each biological replicate, qPCR dual replicates were run
Repeatability (intraassay variation)	E	see Figure S1
Reproducibility (interassay variation, CV)	D	-
Power analysis	D	-
Statistical methods for results significance	E	For the *in silico* expression profile analysis a nonparametric Kruskal-Wallis test was used to detect statistical difference ($p<0.05$) between samples. The expression of the candidate reference genes was evaluated according to Expósito-Rodríguez et al. (2008), by applying two different statistical approaches: using geNorm v.3.5 software (Vandesompele et al. 2002) and NormFinder software (Andersen et al. 2004). For validation the Cq values of target genes were normalized against the geometric average of a combination of reference genes, followed by ANOVA analysis.
Software (source, version)	E	algorithms incorporated to the fgStatistics software (Di Rienzo, 2009)
C_q or raw data submission with RDML		-

Supplemental Table. MIQE form, qRT-PCR and sample information (only genes included in subsequent analyses).

Gene	Gene symbol	Unigene	Primer sequences	Amplicon length (bp)	Annealing temperature (ºC)	cDNA quantity (ng/µl)	Primer concentration [nM]	Mean PCR efficiency
Arginine decarboxylase[a]	*AaADC*	comp39741_c0_seq1	5'-GGTGGAGGGCTTGGCATC-3'<br>5'- CGAAAACGAGGAGGGAATGG-3'	199	60	10	400	1.861
Catalase[a]	*AaCAT*	comp53056_c0_seq2	5'-GCTTTTGGAGGACTATCACC-3'<br>5'-GAGAATCGCACAATAACGGG-3'	192	60	10	400	1.840
Cyclophilin	*AaCYP*	comp39853_c0_seq1	5'-GAAAGTTGTTGTTGAAGATTGCGGC-3'<br>5'-CGTAAACCCTCACAGTAGAAAACC-3'	153	60	10	400	1.909
Elongation Factor 1α	*AaEF-1α*	comp52960_c0_seq8	5'-GATGACGATGATGAGGTTTTACTG-3'<br>5'-CGGCATAATGATTCCACAGC-3'	164	60	10	400	1.885
F-BOX family protein	*AaFBOX*	comp48365_c0_seq1	5'-CGTCCCCAAATCTTCTCTTCC-3'<br>5'-GCAAAAGCGAGTTGTTATCTGATG-3'	196	60	10	400	1.943
Protein phosphatase 2A	*AaPP2A*	comp39762_c0_seq1	5'-GATGAAGGTCAATGTAGAGGG-3'<br>5'-GGTGGGGCTTATTTTGCTTTG-3'	178	60	10	400	1.902
Translational initiation factor 4B	*AaEIF4B-L*	comp50365_c0_seq1	5'-CAGTCGCCTCCTGTCTTG-3'<br>5'-CCGTCGTCTGGTGAAAATG-3'	233	60	10	400	1.876
Trehalose -6-phosphate synthase[a]	*AaTPS3*	comp52170_c0_seq5	5'-CGATGAATGTAGCCCTCACTATGC-3'<br>5'-CTCAATCCAAATCCAATACCCCAGC-3'	178	60	10	400	1.870
Ubiquitin-conjugating enzyme 21	*AaUBC21*	comp42656_c0_seq3	5'-CTCTGGTGATAATCGTGGG-3'<br>5'-CACTGGCAGCAAATGGTTG-3'	185	60	10	400	1.884
UDP-glucose pyrophosphorylase[a]	*AaUGP*	comp39733_c0_seq1	5'-GAAGTTGTGGTTCCCTATC-3'<br>5'-CTCTGCTATTGTATTTGTCGTTGAG-3'	214	60	10	400	1.864

[a] Genes used for reference gene validation.

Supplemental Table. MIQE form, qRT-PCR and sample information.

	sample #	Mass for RNA extraction (mg)	[RNA] (ng/ul)	RNA volume (ul)	260/280	260/230	ng RNA	ng RNA/mg FW
**GZE**	**1**	960	**994.2**	30	2.12	2.41	29826	31
	**2**	900	**796.1**	30	2.13	2.34	23883	27
	**3**	1100	**805**	30	2.12	2.30	24150	22
**CZE**	**1**	802	**966.82**	30	1.9	1.95	29005	36
	**2**	700	**566.86**	30	1.94	1.88	17006	24
	**3**	821	**512.46**	30	1.97	1.92	15374	19
**CZE MG**	**1**	940	**3511.7**	30	1.82	2.13	105351	112
	**2**	944	**3672.3**	30	1.69	2.01	110169	117
	**3**	930	**3800**	30	1.69	1.97	114000	123
**SE1**	**1**	74	**2269.1**	30	2.02	2.08	68073	920
	**2**	100	**1448.9**	30	2.05	1.80	43467	435
	**3**	109	**2013.2**	30	2.04	2.16	60396	554
**SE6**	**1**	100	**1032**	30	2.08	2.41	30960	310
	**2**	93	**731.9**	30	2.08	2.2	21957	236
	**3**	110	**1063.9**	30	2.08	2.41	31917	290
**S1M**	**1**	89	**616.8**	30	2.1	2.01	18504	208
	**2**	98	**772.5**	30	2.09	1.77	23175	236
	**3**	94	**671.5**	30	2.08	2.00	20145	214
**ACICULAS**	**1**	289	**252.3**	30	1.84	1.74	7569	26
	**2**	298	**244.3**	30	1.85	1.86	7329	25
	**3**	294	**238.9**	30	1.86	1.75	7167	24
